# A brief overview of managing prepubertal children who have experienced sexual abuse

**DOI:** 10.4102/safp.v68i1.6254

**Published:** 2026-06-08

**Authors:** Marianne Kotzé, Chika K. Egenasi, Mathew O. Benedict

**Affiliations:** 1 Forensic Medicine, Independent Clinical Forensic Consultant, Cape Town, South Africa; 2 Department of Family Medicine, Faculty of Health Sciences, University of the Free State, Bloemfontein, South Africa

**Keywords:** children, sexual abuse, examination, forensic, position

## Abstract

In South Africa, undergraduate medical teaching does not prepare medical and nursing practitioners adequately for the clinical forensic management of patients who have experienced child sexual abuse. Clinicians might be apprehensive when confronted with children who may have experienced sexual abuse. Their insecurities arise from a perceived lack of knowledge and the time-consuming nature of the legal procedures. The high prevalence of the sexual abuse of children demands the involvement of the medical profession. This article aims to allay uncertainty and address the unnecessary barriers to the provision of service and support to vulnerable pre-pubertal children who are victims of abuse.

## Introduction

Child sexual abuse (CSA) is a complex public health challenge.^1^ When addressing CSA, the best interests of the child take precedence above all else. Safeguarding children’s emotional and physical wellbeing must be prioritised, even above the forensic investigation.^2^ In South Africa, 1 out of 3 children suffers from some form of sexual abuse.^3^ Medical practitioners’ avoidance of the issue^4^ might disadvantage a significant number of their paediatric patients.

## Nature of child sexual abuse

Child sexual abuse is the involvement of a child in sexual activity in circumstances where there is an imbalance in power, which includes monetary, physical strength, emotional, age and intellectual power.^5^ Adults or other children perpetrate the sexual activity, mostly family members or acquaintances.^5,6^ They may be male or female from all socio-economic spheres.^7^ Definitions, terminologies and recognition of CSA vary across cultural, legal and social contexts, as norms around childhood, sexuality, consent and acceptable adult–child interactions are socially constructed and embedded within differing legal frameworks.^8^ The sexual activity comprises acts of non-contact sexual abuse, contact non-penetrative sexual acts or sexual penetration.^9,10,11^ The perpetrators exert power utilising non-physical coercion such as bribery, threats, seduction or provision of the material or emotional needs of the children. A minority uses physical force.^12^

## Consent for the medical examination

Children 12 years and older give consent for their medical examinations when they are of sufficient maturity to understand the nature of the consent.^2,11^ For children under 12 or lacking the maturity to understand consent, guardians usually provide consent unless they are unavailable, deceased, suspected offenders, have a mental disability, cannot or will not give timely consent.^11^ In such cases, under Section 335B of the *Criminal Procedure Act*, if a magistrate’s consent cannot be obtained, the investigating officer must declare this under oath, the medical practitioner must confirm under oath that the examination is urgent, and only then may the commissioned police officer or the person in charge of the local police station, grant written consent.^13^ Under no circumstances should a child be compelled to undergo the collection of forensic evidence.^14^

## Timing of the examination

The conduct of the examination depends on medical, safety, emotional and forensic urgency. Emergency examinations must be carried out without delay, while urgent or ‘as soon as possible’ examinations should be completed within 1–7 days.^15,16^ In settings where specialised forensic services are not immediately available, initial clinical assessment and essential management should be provided, with urgent referral to specialised services within the recommended time frame. The Child Advocacy Centre (CAC) model exemplifies a multidisciplinary, child-centred approach, integrating investigative, medical and therapeutic services within a single, child-friendly environment to minimise further trauma and support the child’s recovery^17^ (Box 1 and Box 2).

BOX 1 Timing of medical examination of prepubertal children.

**Table:** 

**Timing of Medical Examinations**
***Indications for emergency evaluation***
Medical, psychological, or safety concerns such as acute pain or bleeding, suicidal ideation, or suspected human trafficking
Alleged assault that may have occurred within the previous 72 h (or other state-mandated time interval) necessitating collection of trace evidence for later forensic analysis.
Need for emergency contraception.
Need for postexposure prophylaxis (PEP) for STIs, including human immunodeficiency virus (HIV).
***Indications for urgent evaluation***
Suspected or reported sexual contact occurring within the previous 2 weeks, without emergency medical, psychological, or safety needs identified.
***Indications for nonurgent evaluation***
Disclosure of abuse by a child, sexualised behaviours, sexual abuse suspected by a multidisciplinary team, or family concern for sexual abuse, but contact occurred more than 2 weeks prior without emergency medical, psychological, or safety needs identified.
***Indications for follow-up evaluation***
Findings on the initial examination are unclear or questionable, necessitating re-evaluation.
Further testing for STIs not identified or treated during the initial examination.
Documentation of healing/resolution of acute findings
Confirmation of initial examination findings when the initial examination was performed by an examiner who had conducted fewer than 100 such evaluations.

*Source*: Adapted from Adams JA, Kellogg ND, Farst KJ, et al. Updated guidelines for the medical assessment and care of children who may have been sexually abused. J Pediatr Adolesc Gynecol. 2016;29(2):81–87. https://doi.org/10.1016/j.jpag.2015.01.007^15^

STI, sexually transmitted infection.

BOX 2 Positions and techniques of anogenital examination of prepubertal children.

**Table:** 

**Examination positions and techniques**	
***Genital examination of prepubertal child***	
Examination positions	Supine frog-leg or lithotomy position
	Prone knee-chest position
Examination techniques	Labial separation and traction
	Prone knee-chest position with gluteal lift
	Speculum examinations are not indicated unless the child is sedated
Confirmatory technique	Floating hymen with water or saline
	Prone knee-chest position with gluteal lift
***Anal examination of prepubertal child***	
Examination positions (in order of preference)	Supine knee-chest
	Prone knee-chest
	Lateral decubitus
Examination technique	Buttock separation
	Prone knee-chest position with gluteal lift
Confirmatory technique	Reassess after bowel movement, ambulating, or alternate position

*Source*: Adapted from Adams JA, Kellogg ND, Farst KJ, et al. Updated guidelines for the medical assessment and care of children who may have been sexually abused. J Pediatr Adolesc Gynecol. 2016;29(2):81–87. https://doi.org/10.1016/j.jpag.2015.01.00715^15^

## Holistic care of child victims of sexual abuse

The care of sexually abused children extends beyond forensic needs, prioritising the child’s best interests. Avoiding secondary trauma is of paramount importance.^18^ Children should not be coerced into examinations, as control tactics echo those used by abusers. Offering a toy or gift to comfort them is appropriate if it is unconditional. A child’s sense of control is essential; if they are uncomfortable and no medical urgency exists, the examination should be postponed until they feel safe and comfortable.

## History and preparation for the physical examination

Medical history taking in CSA cases involves symptom-focused questions aimed at identifying injuries, infections and immediate health and safeguarding needs to guide clinical care. Forensic interviewing, in contrast, uses open-ended, non-leading questioning to obtain a detailed and legally reliable account of events for investigative and judicial purposes. The HCP must balance the two purposes.

Children’s disclosure of CSA is generally delayed and incremental.^18^ Conversations with medical practitioners may add detail to other interviews,^19,20^ helping to differentiate symptoms like urogenital discomfort and bleeding from trauma.^21,22,23^

All questions posed to the child and their verbatim responses must be carefully documented, and examiners should avoid making assumptions about the child’s meaning. Where appropriate, interviews may be audio- or video-recorded to enhance accuracy.^24,25^ Conversations begin with small talk, then transition into open-ended questions about the purpose of the visit and details of the incident. Age-appropriate questions guide ‘where, when, who, and how’, ensuring clarity without leading suggestions.^26^ Preparation includes explaining that the examination is painless and using dolls or toys to illustrate positions such a frog ‘frog leg’ and a cat stretching out ‘prone knee-chest’ for familiarity and comfort.

## General examination

When conducting a medical examination for a child with suspected sexual abuse, it is essential to ensure that the physical environment is safe, private and child-centred, that a chaperone is present to protect the child’s dignity and support the process and that the timing of the examination is determined by clinical need and the urgency of forensic evidence collection.^27^

A thorough, non-threatening general examination is crucial to building trust and easing children into the anogenital examination. By integrating anogenital and extragenital assessments, the examination normalises all body areas and avoids placing undue emphasis on the anogenital region. The general examination helps identify necessary medical interventions. It establishes a differential diagnosis, guiding decisions on the anogenital exam, further investigations and forensic evidence collection based on the child’s history and findings. Clinicians should ensure that medical forensic examinations are clearly indicated and minimally intrusive, balancing clinical benefit with the need to prevent additional distress and secondary trauma in the child.^10^

## Anogenital examination

Children’s right to refuse an examination should be honoured if no urgent medical need exists. Proper preparation and clear explanations generally help children feel comfortable. Examiners should guide children through each step and avoid unexpected touch. If children remain tense, the examination becomes technically challenging, and the findings are unreliable.

## Positions of the anogenital examination of prepubertal children

It is recommended to utilise multiple examination positions to distinguish structural abnormalities from normal anatomical variants (Box 2).^28,29^.

### Frog-leg position

The frog-leg position involves placing a child in a supine position with hip flexion and abduction. The outer aspects of the thighs rest on the surface of the examination couch. In this position, the external genitalia are visible.

### Prone knee-chest position

To guide a child into a prone knee-chest (PKC) position, they kneel with knees slightly apart, bending forward to rest their ear flat on the examination couch, facing the support person. Arms lie flat beside the head (not resting on the elbows), and the chest is lowered onto the couch to relax the spine. Proper alignment (head on the ear, arms flat, hips at a 90° angle). In this position, the vagina opens under gravity. The posterior rim of the hymen smooths out, allowing for a more accurate diagnosis of anatomical variation and previous trauma of the anus and female genitalia. The anus is visible without touching, and testing for dynamic anal dilatation can be performed.

### Supine knee-chest position

In the supine knee-chest (SKC) position, they lie on their backs, knees approaching the chest as comfortably as possible. The SKC position is an excellent examination position for the anal examination of boys since their genitalia are examined in other positions.

## Techniques for the anogenital examination of prepubertal children

### Labial separation

In the frog-leg position, the labia are gently separated with the touch of the labia majora and lateral movement.The posterior commissure, fossa navicularis, vestibule and hymen become visible, but the margins of the hymen cannot be adequately visualised.^14,28^

### Labial traction

The labia majora is held bilaterally between the thumbs and forefingers and pulled in the examiner’s direction. Gentle manipulation of the labia allows visualisation of the hymen. The hymenal opening may be better visualised, but the vagina is a potential space, and the edge of the hymen is still relaxed.^14,28^

### Floating of the hymen with water or saline

The labia minora may adhere, making visualisation difficult. Dropping water at body temperature onto the genitalia breaks the adhesion and allows the structures to float.^14,28^

### Gluteal separation

Gluteal separation can be done in the frog leg, PKC or SKC and lateral recumbent positions. The best visualisation of the anus is achieved in the PKC and SKC positions. The technique is mainly utilised to test for dynamic anal dilatation.^14,28^

### Gluteal lift

In the PKC position, the examiner places his hands laterally to the superior aspects of the labia majora and gently pulls upward and outward. The vagina opens under gravitational force. The posterior hymenal rim, the location where structural abnormalities of the hymen occur, is smoothed out, the margins of the hymen can be visualised, and the differentiation between abnormalities and normal anatomical variation can be assessed. The anus is visualised excellently with this position and technique.^14,28^

## Collection of evidence

Forensic evidence collection is ideally performed within 24 h for prepubertal children and within 72 h for adolescents. In some young children, evidence collection beyond 24 h may still be considered when appropriate.^15^ Deoxyribonucleic acid (DNA) retrieval from prepubertal girls’ bodies is extremely rare, with a higher likelihood of evidence recovery from clothing or bed linen.^22,30,31,32,33^ Because of the genital sensitivity and children’s intolerance to hymenal and vestibular contact, evidence collection must be handled with particular care and sensitivity.

## Screening for sexually transmitted infections

Physicians should consider sexually transmitted infection (STI) screening in cases involving oral and anogenital penetration, abuse by a stranger, a person known or suspected to have an STI or high infection risk or when a household member has an STI.^30^ Screening is also advised if the child shows STI signs, lives in a high-STI area, has vaginal discharge or has previously been diagnosed with an STI.^31,32^ Diagnosis must meet international quality standards, using confirming culture or serology.^33^

## Examining under sedation or anaesthesia

Sedation or anaesthesia for examinations should be limited to cases with specific medical indications, such as acute bleeding, lower abdominal tenderness, severe injuries or foreign object use, and not solely for forensic purposes, as anaesthesia carries rare but serious risks.^34,35^ For non-acute cases, over 90% of exams in prepubertal children yield normal findings, and the probability of DNA recovery is close to zero.^22,30,31^ Procedures with no health benefit expose healthcare providers to legal liability.^36^

## Interpretation of assessment

### History

A clear history with details of time, place, sound, smell, touch and sensation and an imbalance of power is suggestive of CSA.^5,22,37^

### Physical findings

Congenital absence of the hymen does not occur as an isolated anomaly in otherwise normally developed genitalia.^38^ See Box 3 for more information.

BOX 3 2023 updated approach to interpretation of medical findings in suspected child sexual abuse.

**Table:** 

**Section 1: Physical findings** **A. Findings documented in newborns or commonly seen in non-abused children** * These findings are normal and are unrelated to a child’s disclosure of sexual abuse.	
**Normal variants**	
1	**Hymenal variations** a) **Annular**: hymenal tissue present all around the vaginal opening, including at the 12 o’clock location b) **Crescentic hymen**: hymenal tissue is absent at some point above the 3 to 9 o’clock locations c) **Imperforate hymen**: hymen with no opening d) **Micro-perforate hymen**: hymen with one or more small openings e) **Septate hymen**: hymen with one or more septae across the opening f) **Redundant hymen**: hymen with multiple flaps, folding over each other g) Hymen with a tag of tissue on the rim h) Hymen with mounds or bumps on the rim at any location i) Any notch or cleft of the hymen (regardless of depth) above the 3 and 9 o’clock location j) A notch or cleft in the hymen, at or below the 3 o’clock or 9 o’clock location that does not extend nearly to the base of the hymen k) The smooth posterior rim of the hymen, which appears to be relatively narrow along the entire rim, may give the appearance of an enlarged opening. l) Asymmetry in the width of the posterior hymenal rim
2	Periurethral or vestibular bands
3	Intravaginal ridge(s) or column(s)
4	External ridge on the hymen
5	Diastasis ani (smooth area)
6	Perianal skin tag(s)
7	Hyperpigmentation of the hymen, labia minora or perianal tissues
8	Dilation of the urethral opening
9	Normal midline anatomic features a) Groove in the fossa, seen in early adolescence b) Failure of midline fusion (also called perineal groove) c) Median raphe d) Linea vestibularis (midline avascular area)
10	Visualisation of the pectinate/dentate line at the juncture of the anoderm and rectal mucosa, seen when the anus is fully dilated, as with the passage or presence of flatus or stool in the anal canal
11	Reflex anal dilation that occurs during examination manoeuvres, such as traction applied to perianal tissues or positioning the patient, particularly in prone or supine knee-chest positions
12	Anal dilation, causing visualisation of the dentate/pectinate line, anal columns and/or anal crypts, any of which may be mistaken for anal laceration or abrasion
**B. Findings commonly caused by conditions other than trauma or sexual contact** **These findings require that a differential diagnosis be considered, as each may have several different causes**	
13	Erythema, inflammation, fissuring and/or maceration of the perianal, perineal or vulvar tissues related to poor hygiene or other irritant dermatitis
14	Increased vascularity of the vestibule and hymen
15	Labial adhesion
16	Friability of the posterior fourchette
17	Vaginal discharge that is not associated with a sexually transmitted infection
18	Anal fissures
19	Venous congestion or venous pooling in the perianal area
20	Complete and/or immediate anal dilatation in children with pre-disposing conditions, such as current symptoms or history of constipation and/or encopresis or children who are sedated, under anaesthesia or with impaired neuromuscular tone for other reasons
**C. Findings due to other conditions, which can be mistaken for abuse**	
21	Irritative and/or non-infectious: erythema, inflammation and fissuring of the perianal, perineal or vulvar tissues because of irritant dermatitis, including Jacquet’s dermatitis
22	Inflammatory: aphthous ulcers, inflammatory bowel disease (anal fissures and/or prominent anal tags, rectal discharge), Behcet’s disease (painful ulcers)
23	Dermatologic conditions: lichen sclerosis et atrophicus, folliculitis, vitiligo, angiokeratomas and haemangiomas
24	Immunologic causes: pyoderma gangrenosum (painful ulcers)
25	Multifactorial and/or idiopathic: urethral prolapse, rectal prolapse, anal funnelling
26	Post-mortem changes: anal dilatation, red/purple discolouration of the genital structures (including the hymen) from lividity or other rare systemic conditions. Histologic analysis is needed for confirmation.
**D. No expert consensus regarding the degree of significance** These physical findings have been associated with a history of sexual abuse in some studies; but at present, there is no expert consensus as to how much weight they should be given with respect to abuse. Findings 28 and 29 should be confirmed using additional examination positions and/or techniques to ensure they are not normal variants or a finding of residual traumatic injury (finding 38)	
27	Complete and immediate anal dilation with relaxation of the internal as well as external anal sphincters in the absence of other predisposing factors such as constipation, encopresis, sedation, anaesthesia and neuromuscular conditions
28	Notch or cleft in the hymen rim, at or below the 3 o’clock or 9 o’clock location, which extends nearly to the base of the hymen but is not a complete transection. This rare finding should be interpreted with caution unless an acute injury was documented at the same location.
29	Complete cleft and/or suspected transaction to the base of the hymen at the 3 or 9 o’clock location.
**E. Findings caused by trauma** **These findings are highly suggestive of abuse, even in the absence of a disclosure from the child, unless the child and/or caretaker provides a timely and plausible description of accidental anogenital straddle, crush or impalement injury or past surgical interventions that are confirmed from the review of medical records. Findings representing residual and/or healing injuries should be confirmed using additional examination positions and techniques. Isolated, few superficial injuries that appear to be bruises or petechiae should be confirmed as traumatic injuries by showing resolution on follow-up examination. Photographs or video recordings of these findings should be taken, then evaluated and confirmed by an expert in sexual abuse evaluation to ensure accurate diagnosis.**	
**1) Acute trauma to genital and/or anal tissues**	
30	Acute laceration(s) or bruising of labia, penis, scrotum or perineum
31	Acute laceration of the posterior fourchette or vestibule, not involving the hymen
32	Bruising, petechiae or abrasions on the hymen
33	Acute laceration of the hymen of any depth, partial or complete
34	Vaginal laceration
35	Perianal bruising or perianal laceration with exposure of tissues below the dermis
**2) Residual (healing) injuries to genital and/or anal tissues**	
36	Perianal scar (a very rare finding that is difficult to diagnose unless an acute injury was previously documented at the same location)
37	Scar of posterior fourchette or fossa (a very rare finding that is difficult to diagnose unless an acute injury was previously documented at the same location)
38	Healed hymenal transection/complete hymen cleft – a defect in the hymen below the 3–9 o’clock location that extends to or through the base of the hymen, with no hymenal tissue discernible at that location
39	Signs of female genital mutilation (FGM) or cutting, such as loss of part or all of the prepuce (clitoral hood), clitoris, labia minora or labia majora, or vertical linear scar adjacent to the clitoris (Type 4 FGM)
**3) Acute trauma to oral tissues**	
40	Acute oral trauma, such as unexplained injury or petechiae of the lips or palate, particularly near the junction of the hard and soft palate
**Section 2: Infections**	
**A. Infections not related to sexual contact**	
41	Erythema, inflammation, fissuring of perianal, perineal or vulvar tissues because of bacteria, fungus, virus or parasites that are transmitted by non-sexual means, such as Streptococcus Type A or Type B, *Staphylococcus sp*., *Escherichia coli*, Shigella or other gram-negative organisms
42	Genital ulcers caused by viral infections such as the Epstein-Barr Virus
**B. Infections that can be spread by (or are associated with) sexual transmission as well as non-sexual transmission. *Interpretation of these infections may require additional information, such as the mother’s gynaecologic history (HPV), the child’s history of oral lesions (HSV) or the presence of lesions elsewhere on the body (molluscum), which might clarify the likelihood of sexual transmission*.**	
43	Molluscum contagiosum in the genital or anal area. In young children, transmission is most likely non-sexual. Transmission from intimate skin-to-skin contact in the adolescent population has been described.
44	*Condyloma acuminatum* (HPV) in the genital or anal area.
45	Herpes simplex Type 1 or 2 infections in the oral, genital or anal area diagnosed by culture or nucleic acid amplification test
46	Urogenital *Gardnerella vaginalis* (associated with sexual contact but also found in prepubertal and adolescent vaginal flora)
47	Urogenital *Mycoplasma genitalium* or *Ureaplasma urealyticum*; while sexually transmitted in adolescents, the prevalence and transmission of these infections in children are not well understood
**C. Infections caused by sexual contact, if confirmed by appropriate testing and perinatal transmission, have been ruled out**	
48	Genital, rectal or pharyngeal *Neisseria gonorrhoea* infection
49	Syphilis
50	Genital, rectal or pharyngeal *Chlamydia trachomatis* infection
51	*Trichomonas vaginalis* infection isolated from vaginal secretions or urine
52	HIV, if transmission by blood or contaminated needles has been ruled out
**SECTION 3: FINDINGS DIAGNOSTIC OF SEXUAL CONTACT**	
53	Pregnancy
54	Semen identified in forensic specimens taken directly from a child’s body

*Source*: Adams JA, Farst KJ, Kellogg ND. Interpretation of medical findings in suspected child sexual abuse: An update for 2018. J Pediatr Adolesc Gynecol. 2018;31(3):225–231. https://doi.org/10.1016/j.jpag.2017.12.011 and Kellogg ND, Farst KJ, Adams JA. Interpretation of medical findings in suspected child sexual abuse: An update for 2023. Child Abuse Negl. 2023;145:106283. https://doi.org/10.1016/j.chiabu.2023.106283^39,40^

## Conclusion

Child sexual abuse among prepubertal children represents a widespread public health crisis. Inadequate awareness, training and specialised skills among healthcare professionals at the primary care level undermine both effective child protection and judicial outcomes. Deficiencies in undergraduate and postgraduate training further contribute to poor quality documentation and testimony. Healthcare professionals should recognise that they are examining children who have experienced sexual abuse, not the abuse itself; the assessment therefore carries a therapeutic responsibility that prioritises the child’s wellbeing while also fulfilling medico-legal obligations.
